# Spatially-informed interpolation for reconstructing lake area time series using semantic neighborhood correlation

**DOI:** 10.1038/s41598-025-09410-3

**Published:** 2025-07-09

**Authors:** Chen Liu

**Affiliations:** School of Remote Sensing and Information Engineering, Wuhan University, Wuhan, 430079 China

**Keywords:** Lake area interpolation, Remote sensing, Spatiotemporal analysis, Hydrological monitoring, Environmental sciences, Hydrology

## Abstract

Long-term, high-resolution records of lake surface area are essential for characterizing the spatiotemporal dynamics of inland water bodies. Although Synthetic Aperture Radar has substantially improved water extent detection under adverse conditions, optical remote sensing imagery remains the dominant data source owing to its higher spatial resolution. Nevertheless, optical data are frequently compromised by persistent cloud cover and sensor limitations, leading to substantial observational gaps. To effectively address this challenge, this study introduces a novel spatially-informed interpolation method termed Semantic Neighborhood Correlation-based Interpolation (SNCI), which leverages spatial correlations among hydrologically interconnected lakes to reconstruct missing lake area observations. By explicitly modeling the inherent hydrological and climatic coherence among neighboring lakes, SNCI provides robust, accurate, and scalable interpolations even in the presence of extensive temporal data losses. The method was evaluated on monthly lake area data from 54 lakes in the Wuhan region between 2000 and 2020, using the Global Surface Water dataset, and validated against high-resolution Dynamic World observations. Several representative lakes were analyzed in detail to assess SNCI’s robustness across diverse seasonal and interannual conditions. Compared with polynomial fitting, Random Forest, and Long Short-Term Memory, SNCI consistently achieves lower interpolation errors. In the case of East Lake, SNCI reduces mean absolute error by 50.1% and root mean square error by 28.3% relative to the best-performing baseline. Across all lakes, SNCI demonstrates superior accuracy and correlation, particularly under data-sparse conditions. These results underscore SNCI’s potential to enhance lake area reconstruction accuracy and support broader applications in hydrological modeling, environmental monitoring, and climate impact assessment.

**Supplementary Information:**

The online version contains supplementary material available at 10.1038/s41598-025-09410-3.

## Introduction

Water resources play a pivotal role in sustaining ecosystems and ensuring human welfare. Lakes serve as key freshwater reservoirs. They sustain biodiversity, regulate hydrological cycles, and support various socio-economic functions^1–4^. However, these vital aquatic systems are increasingly threatened by global environmental pressures, particularly the accelerating impacts of climate change and rapid human-induced transformations. Rising temperatures, shifting precipitation patterns, urban sprawl, and intensified agricultural practices have collectively driven major changes in lake size, distribution, and ecological integrity^5,6^. Accordingly, accurate and long-term monitoring of lake area dynamics is essential for effective water resource management, disaster risk reduction, and the sustainable governance of aquatic ecosystems^7,8^.

Remote sensing has become the principal tool for monitoring surface water bodies, owing to its broad spatial coverage, temporal consistency, and cost-efficiency. Optical satellite missions such as Landsat and Sentinel-2 have been extensively employed to delineate lake boundaries and monitor their temporal dynamics^9–12^. However, their effectiveness is frequently hindered by persistent cloud cover, snow, and atmospheric interference, particularly in humid or high-altitude regions^13^. To overcome these limitations, Synthetic Aperture Radar (SAR) systems—capable of all-weather, day-and-night imaging—have been increasingly utilized^14,15^. In particular, numerous studies have reported enhanced water detection through the fusion of optical and SAR data^16–18^. Yet, the historical SAR record remains limited in spatial and temporal resolution, rendering it insufficient for reconstructing long-term lake dynamics^19^. These limitations highlight the need for a spatially informed interpolation approach that can reliably reconstruct missing lake area records without relying solely on continuous satellite observations.

Interpolation offers a promising strategy to address these data gaps. However, various interpolation techniques have been extensively applied to reconstruct missing lake area records, and each method carries inherent limitations^20^. Traditional methods, such as Inverse Distance Weighting^21^ and Kriging^22^, rely solely on spatial proximity, ignoring temporal patterns and hydrological relationships between lakes^23^. Conversely, advanced time-series models such as Long Short-Term Memory (LSTM)^24^ or graph neural networks^25^, although powerful, typically require extensive training data and lack explicit spatial semantic modeling, which makes them less interpretable and less applicable in data-scarce scenarios^26^. To address these challenges, this study proposes SNCI, a method that leverages both temporal coherence and spatial semantic dependencies across lakes. Designed to be interpretable and computationally lightweight, SNCI operates without requiring large labeled datasets, making it well-suited for large-scale historical interpolation tasks under conditions of significant data loss.

SNCI builds on the idea that lakes within the same hydrological or climatic region often fluctuate synchronously^27^. This insight is supported by Tobler’s First Law of Geography, which posits that geographically proximate features often share environmental and hydrological characteristics, leading to correlated temporal dynamics^28,29^. However, spatial proximity alone does not universally guarantee consistent lake behavior. According to the Law of Spatial Heterogeneity^30^, nearby lakes may still differ substantially due to local variations in topography, land use, catchment attributes, or human interventions. To robustly address this complexity, SNCI estimates missing lake area values based on a weighted aggregation of multiple neighboring lakes with demonstrated temporal correlation. This strategy, inspired by geographically adaptive techniques such as Geographically Weighted Regression^31^, enables SNCI to adaptively reflect regional hydrological variability while avoiding overreliance on single references. Unlike conventional interpolation approaches that either ignore spatial interactions or assume global stationarity, SNCI balances interpretability, spatial nuance, and computational efficiency.

To validate the effectiveness of SNCI, its performance was compared with multiple benchmark methods—ranging from traditional interpolators to learning-based models—using multi-year lake observations from the Wuhan region, including detailed analyses of four representative lakes with diverse hydrological characteristics. The remainder of this paper is organized as follows: “Study area and data section” describes the study area and data sources. “Methodology” section details the SNCI methodology, including outlier removal, spatial correlation analysis, the interpolation framework, and baseline methods. “Results” section presents experimental results and comparative evaluations. “Discussion” section discusses key findings, implications, and limitations. Finally, “Conclusion” section concludes the study and outlines future research directions.

## Study area and data

### Study area

**Fig. 1:**
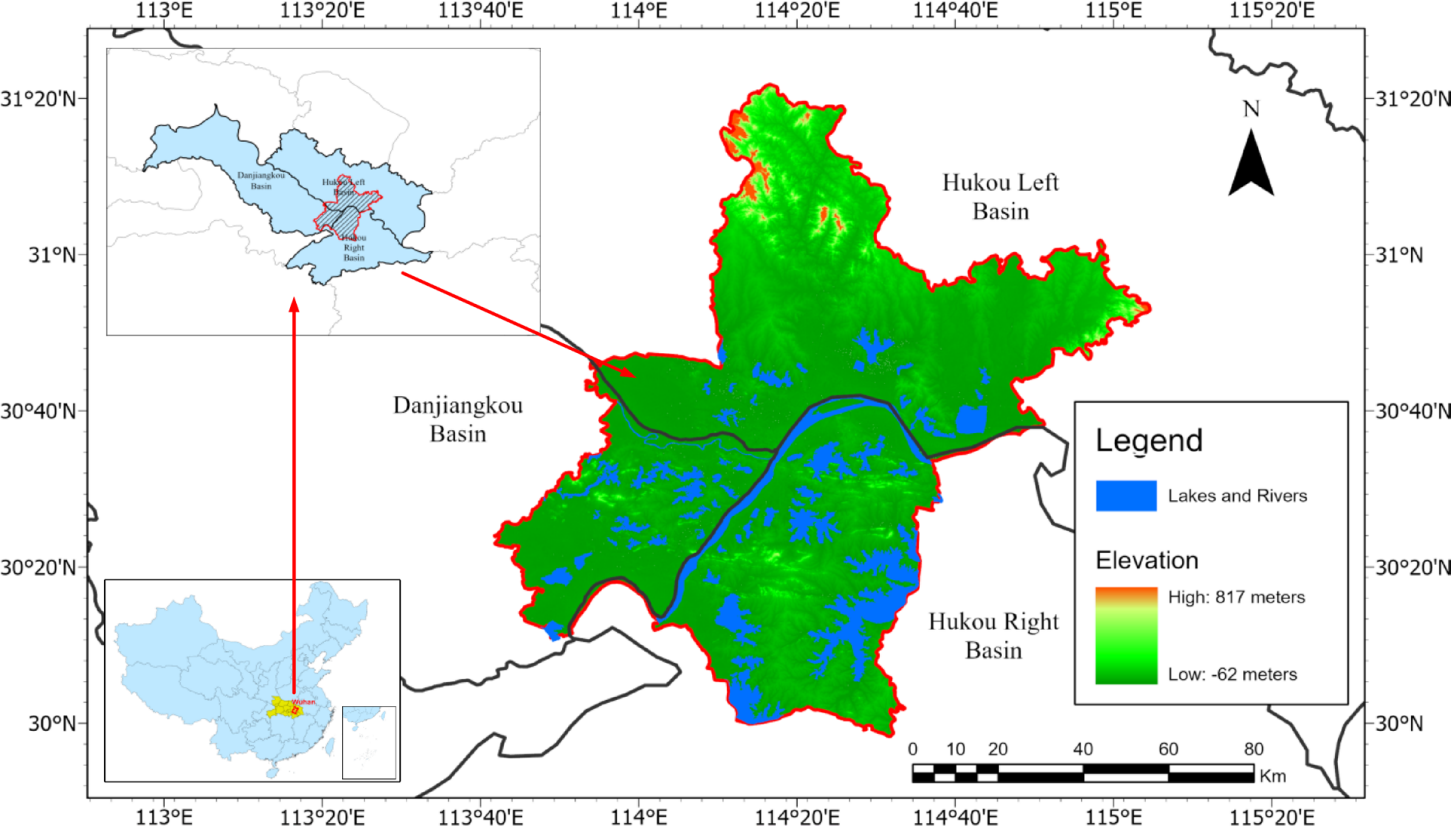
Geographic location and hydrological structure of the study area. The region includes three Basins and features complex hydrological connectivity. Elevation data were derived from the SRTM digital elevation model^34^, while lake and river bodies were extracted from satellite observations. The red boundaries denote the spatial extent analyzed using the SNCI method.

Wuhan, located in central China (113°41′–115°05′ E, 29°58′–31°22′ N), is shown in Fig. 1. As one of China’s ten largest cities, it has a population exceeding 13.5 million. Its location at the confluence of the Yangtze and Han Rivers makes it a critical hydrological node in central China. The city lies at the intersection of three major river basins and is widely known as the “City of Hundred Lakes,” with more than 160 lakes collectively covering approximately 25% of the urban landscape^32^. In recent decades, rapid urbanization has significantly altered the hydrological regime and reshaped lake distributions, highlighting the urgent need for reliable lake monitoring strategies^33^. Furthermore, persistent cloud cover presents additional challenges for optical remote sensing, reinforcing Wuhan’s suitability as a representative case study for evaluating the performance of the proposed SNCI interpolation method.

### Global surface water dataset

The Global Surface Water (GSW) dataset, developed by the European Union’s Joint Research Centre, provides globally consistent surface water extent maps derived from Landsat imagery at a spatial resolution of 30 m. It includes both monthly dynamics and long-term occurrence statistics, spanning 1984 to 2021^35^. In this study, monthly lake surface areas derived from the GSW dataset serve as the primary input for interpolation, while the permanent water extent product defines historical baselines and assists in outlier detection. Its global coverage, methodological consistency, and long-term continuity make the GSW dataset particularly well-suited for monitoring lake dynamics at large spatial scales over multi-decadal periods.

### Dynamic world dataset

The Dynamic World (DW) dataset, co-developed by Google and the World Resources Institute, provides near-real-time global land cover classifications from Sentinel-2 imagery, with a spatial resolution of 10 m and an update frequency of approximately 2–5 days^36,37^. Its high spatial and temporal resolution enables more accurate characterization of dynamic surface water features, outperforming traditional optical datasets in capturing short-term fluctuations. In this study, the DW dataset was used as an independent reference to validate interpolated lake areas from 2017 to 2020. Monthly surface areas were generated by temporally aggregating frequent observations, with further details provided in Sect. 3.5.

### China’s surface water bodies, large dams, reservoirs, and lakes dataset

The China-LDRL dataset, compiled by^38^, delineates the boundaries of over 3,000 large lakes, reservoirs, and dams across China using high-resolution Landsat and Google Earth imagery. It includes lakes larger than 1 km² and serves as a key geographical reference for lake area estimation in this study. To address potential underestimation due to shoreline variability and floodplain dynamics, a 50-meter buffer was applied to each lake polygon, following the approach of^39^. This strategy enhances the consistency and representativeness of area estimates, especially during high-water periods.

Table 1 provides a summary of the key characteristics of the datasets used in this study. Both the DW and GSW datasets were processed using Google Earth Engine (GEE), a cloud-based geospatial analysis platform widely adopted for large-scale environmental monitoring due to its computational efficiency^40,41^. Specifically, monthly lake area time series from 2000 to 2020 were extracted from the GSW dataset, whereas the DW dataset was used as an independent reference for validation during 2017–2020. All lake extents were projected onto the WGS84 coordinate system to ensure spatial consistency across datasets.

**Table 1:** Key specifications of the datasets used in this study.

Dataset name	Source	Spatial Resolution	Update Frequency	Description
Global surface water (GSW)	Joint Research Centre	30 m	Monthly	Global distribution of surface water bodies
Dynamic world (DW)	Google & World Resources Institute	10 m	2–5 days	Near-real-time land cover classification
China-LDRL	Wang et al. (2022)	30 m	Single-date (2019)	Boundaries of lakes and reservoirs in China

### Study lake selection

To better illustrate the strengths and limitations of the SNCI interpolation framework, four representative waterbodies—East Lake, South Lake, West Lake, and Five Lake—were selected from the full set of 54 lakes in the Wuhan region for detailed visualization and case-study analysis. Their contrasting hydrological settings (urban, suburban, and rural), surface-area ranges, and proportions of missing data provide a rigorous and balanced test bed for evaluating the robustness of SNCI under diverse spatiotemporal conditions.

Table 2 summarizes the key attributes of these lakes, including their area variability, Pearson correlation coefficients with the four most temporally similar neighboring lakes, and data availability over the study period.

**Table 2:** Essential parameters for selected lakes.

Lake name	Area range (km^2^)	Correlation coefficients	Data availability (%)	Urbanization level
East Lake	27.6–29.8	0.91, 0.87, 0.83, 0.81	87.1	Urban
South Lake	7.2–7.9	0.82, 0.76, 0.75, 0.69	81.7	Urban
West Lake	6.2–16.1	0.86, 0.85, 0.79, 0.73	79.2	Suburban
Five Lake	7.1–44.5	0.54, 0.38, 0.35, 0.31	79.2	Rural

For each target lake, the four neighboring lakes with the highest Pearson correlation coefficients were selected as reference lakes for SNCI interpolation. Correlation values correspond to the top four correlated lakes. Data availability was calculated over the period 2000–2020 based on valid monthly observations.

**Fig. 2:**
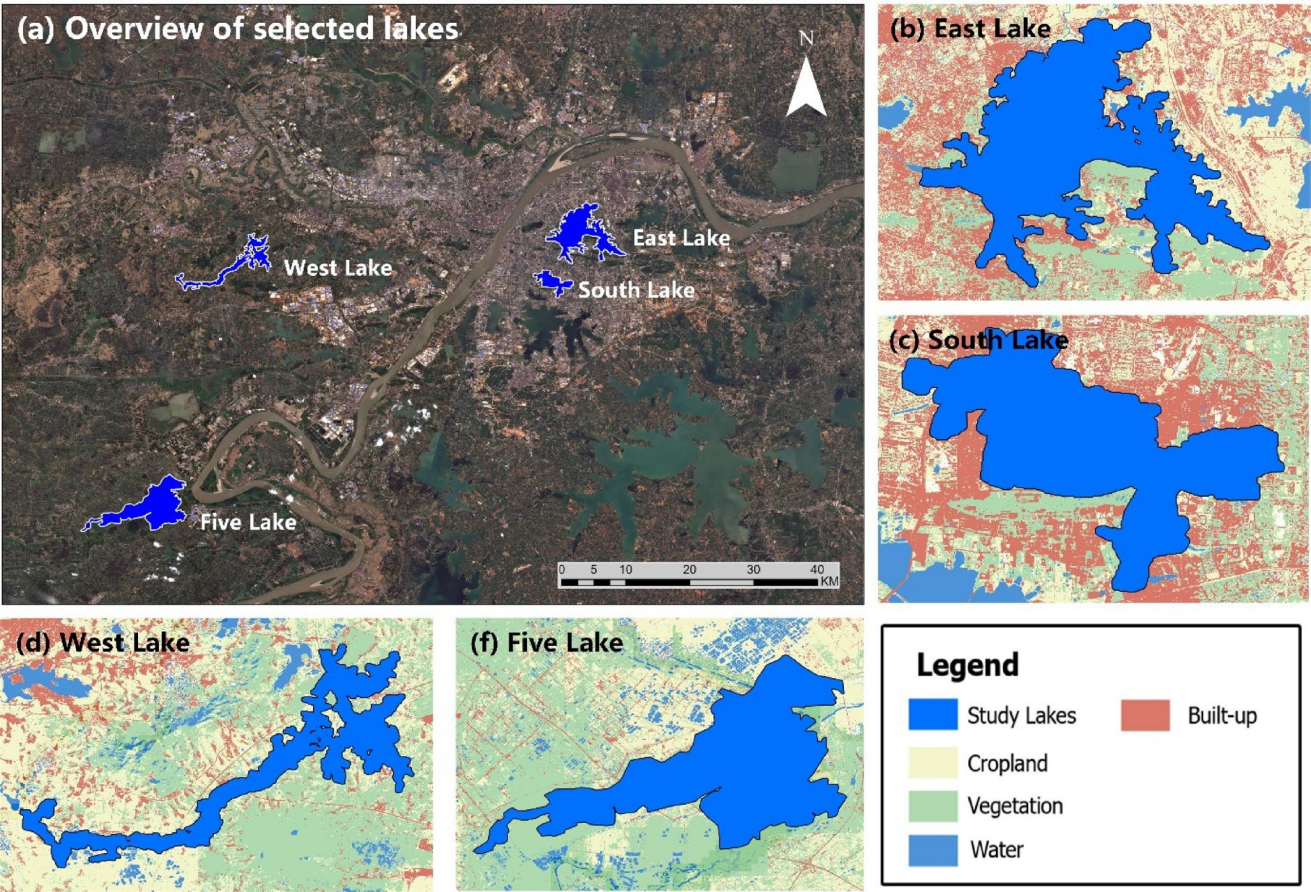
Geographic distribution, catchment boundaries, and surrounding land-use patterns of four selected lakes. (**a**) shows the locations of East Lake, South Lake, West Lake, and Five Lake, overlaid on a Sentinel-2 satellite image derived and visualized using Google Earth Engine (https://earthengine.google.com). Subfigures (**b**)–(**f**) present each individual lake along with its surrounding land cover types, derived from the SinoLC-1 land cover dataset^42^.

Figure 2 locates the four lakes within the regional hydrological network and shows their maximum extents, catchment boundaries, and surrounding land-use patterns derived from Sentinel-2 imagery and the SinoLC-1 land-cover map^42^. East Lake and South Lake are embedded in densely built-up urban environments; West Lake occupies an urban–rural fringe dominated by farmland and natural vegetation; and Five Lake lies in a largely undeveloped wetland–agricultural landscape, exhibiting the greatest hydrological variability. These spatial patterns are illustrated in Fig. 2, which maps the locations, catchments, and land cover types of the selected lakes.

## Methodology

### Overview of workflow

The primary objective of this study is to reconstruct reliable and continuous records of lake surface area by addressing frequent data gaps caused by persistent cloud cover and sensor-related limitations inherent in optical satellite imagery. Conventional temporal interpolation methods often yield suboptimal results due to their failure to incorporate spatial correlations among neighboring lakes. To address this limitation, a three-stage interpolation framework is introduced to explicitly exploit spatiotemporal relationships for enhanced reconstruction accuracy.

**Fig. 3:**
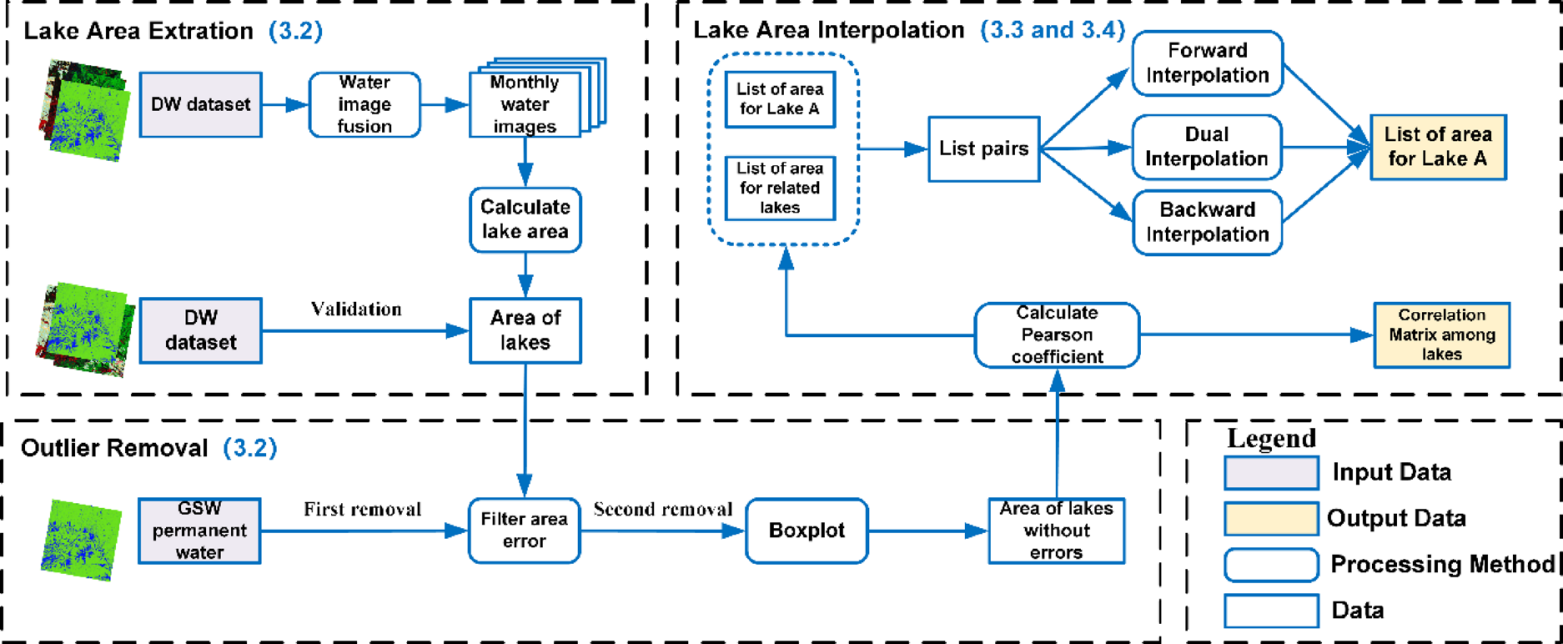
Schematic workflow of the proposed SNCI-based lake area reconstruction framework. The diagram illustrates the three-stage process comprising lake area extraction and outlier removal, neighborhood correlation analysis, and SNCI-based interpolation.

The workflow of the proposed SNCI-based interpolation method is illustrated in Fig. 3. The first stage (Lake Area Extraction and Outlier Removal, detailed in Sect. 3.2) involves deriving monthly lake area datasets and ensuring data reliability through rigorous validation and systematic outlier removal procedures. The second stage (Neighborhood Correlation Calculation, Sect. 3.3) quantitatively assesses the spatiotemporal correlations among lakes, thus establishing robust inter-lake relationships based on their historical temporal dynamics. The third stage (SNCI-based Interpolation, Sect. 3.4) utilizes these quantified correlations to interpolate missing lake area values effectively, thereby ensuring spatially coherent and temporally consistent results.

### Lake area extraction and outlier removal

Accurate estimation of lake surface area is critical for subsequent interpolation and hydrological analysis. Monthly lake area time series were derived from the GSW dataset’s Monthly Water History product, which provides 30-meter resolution classifications based on Landsat imagery^35^. Only pixels labeled as water (value = 2) were included in area calculations.

Lake boundaries were defined using the China-LDRL dataset^38^, which delineates lakes larger than 1 km². To account for shoreline variability and seasonal inundation, each polygon was expanded by a 50-meter buffer^43^. Monthly surface areas from 2000 to 2020 were estimated by counting valid water pixels within the buffered boundaries. All datasets were reprojected to the WGS84 coordinate system to ensure spatial consistency.

To ensure data reliability, a two-step outlier removal strategy was employed, with each lake treated as an independent analysis unit. That is, outliers were identified based on lake-level time series of total surface area, not individual pixel values.

*Step 1* Physical Outlier Removal Based on Permanent Water Extent.

The occurrence band from GSW, indicating water frequency (1984–2021), was used to define a physical baseline. Pixels with > 50% occurrence were classified as permanent water. For each lake, the minimum plausible monthly area was set to the total number of permanent water pixels. Monthly estimates falling below this threshold were considered physically implausible and removed. This threshold was based on a physically informed rule rather than a statistical test. In this context, “significantly lower” refers to values that fall below the long-term observed extent of permanent water, and does not imply statistical significance.

*Step 2* Statistical Outlier Removal via Boxplot Analysis.

Following physical filtering, remaining values were screened for statistical anomalies using the interquartile range (IQR) method^44,45^. Observations outside $\:[Q1\:-\:1.5\times\:IQR,\:Q3\:+\:1.5\times\:IQR]$ were flagged as outliers and excluded, again evaluated at the lake level.

This dual-filtering approach enhances both the physical plausibility and statistical robustness of the lake area time series, forming a reliable foundation for subsequent SNCI-based interpolation.

### Neighborhood correlations calculation

To support the SNCI framework, inter-lake temporal correlations were computed to identify semantically relevant neighbors for interpolation. Specifically, Pearson correlation coefficients were calculated between valid monthly area time series of all lake pairs across the study domain. For each target lake with missing values, neighboring lakes with correlation coefficients exceeding 0.4 were selected as candidate references. This empirically determined threshold represents a moderate correlation strength, balancing inclusion and temporal coherence.

The degree of temporal synchrony between lakes was quantified using the Pearson correlation coefficient, a widely used statistical metric for measuring the strength of linear relationships between two variables^46^. For two lakes $\:X$ and $\:Y$, the Pearson correlation coefficient $\:P$ is calculated as:

$$\:P=\frac{cov(X,Y)}{{\sigma\:}_{X}{\sigma\:}_{Y}}$$

where the covariance $\:cov(X,Y)$ is defined as:

$$cov\left( {X,Y} \right) = \frac{{\mathop \sum \nolimits_{{i = 1}}^{n} \left( {x_{i} - \bar{x}} \right) \times \left( {y_{i} - \bar{y}} \right)}}{{n - 1}}$$

and the standard deviations $\:{\sigma\:}_{X}$ and $\:{\sigma\:}_{Y}\:$are calculated by:

$$\sigma _{X} = \sqrt {\frac{{\mathop \sum \nolimits_{{i = 1}}^{n} \left( {x_{i} - \bar{x}} \right)^{2} }}{{n - 1}}} ,\sigma _{Y} = \sqrt {\frac{{\mathop \sum \nolimits_{{i = 1}}^{n} \left( {y_{i} - \bar{y}} \right)^{2} }}{{n - 1}}}$$

Here, $\:{x}_{i}$ and $\:{y}_{i}$ denote the lake area observations at the $\:i$-th month for lakes $\:X$ and $\:Y$, respectively; $\:\overline{x}$ and ​$\:\overline{y}$ are their corresponding means, and $\:n\:$is the total number of monthly observations.

To ensure robustness against noise and missing data, only valid (non-outlier) months were included. Moreover, dynamic alignment was applied to each pairwise time series, retaining only the intersection of months with valid data in both lakes. This ensures that each computed Pearson coefficient reflects genuine temporal synchrony based on shared, high-quality observations.

The resulting correlation coefficients serve as weights in the SNCI interpolation scheme, assigning greater influence to lakes exhibiting stronger temporal correlation with the target lake. This strategy prioritizes spatially and semantically relevant neighbors, enhancing both the physical plausibility and statistical robustness of the interpolated results.

### Interpolation based on neighborhood correlations

The SNCI method is grounded in the assumption that hydrologically or climatically connected lakes exhibit synchronous fluctuations in surface area. Lakes within the same watershed, for instance, often undergo simultaneous expansion or contraction in response to regional precipitation or evaporation patterns. These correlations enable the use of neighboring lake dynamics to infer missing observations in the target lake.

**Fig. 4:**
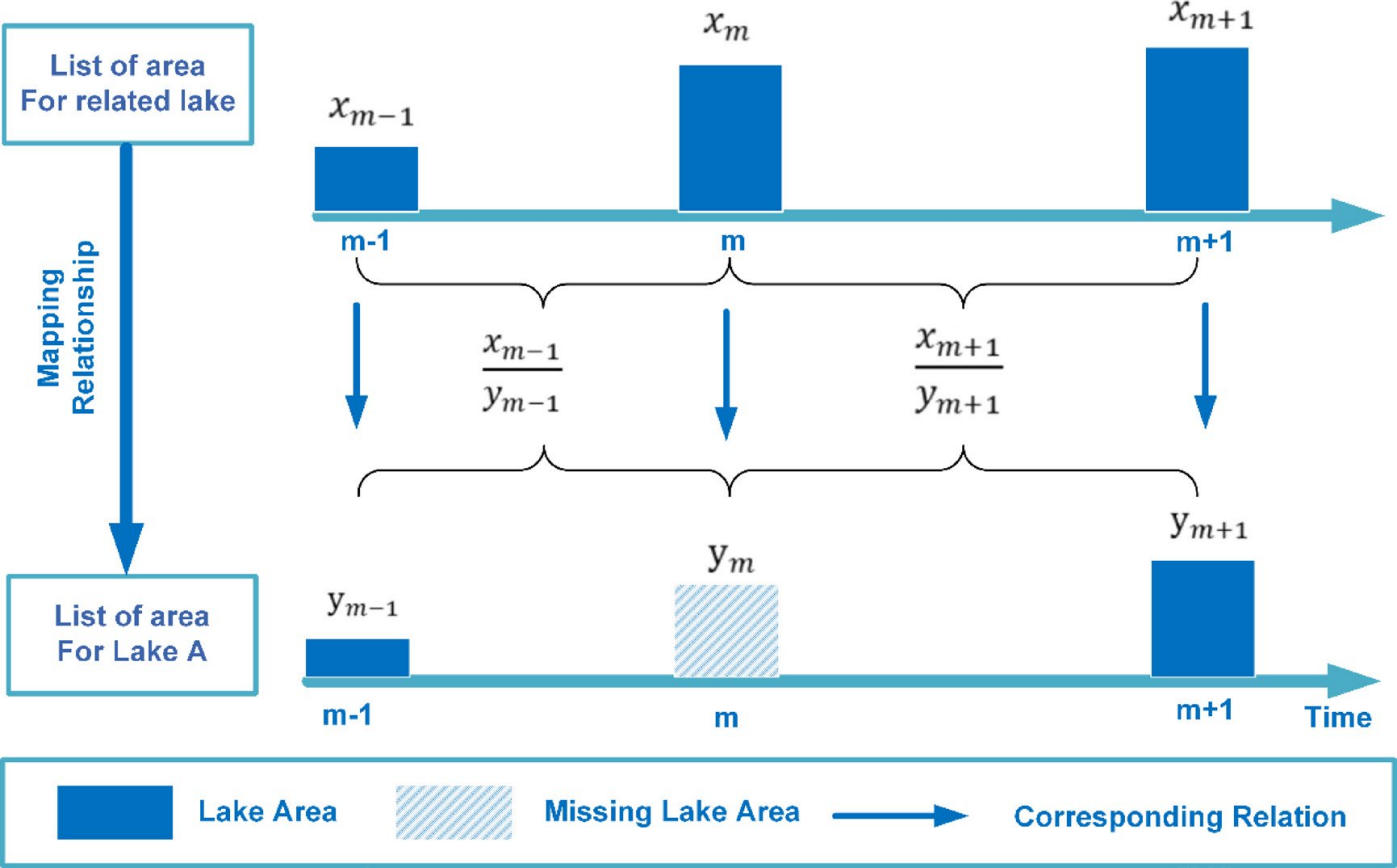
Schematic of the SNCI principle: missing values for a target lake are estimated using observations from temporally correlated neighboring lakes.

Figure 4 schematically illustrates the principle underlying the SNCI method. Consider a target lake with a missing observation $\:{y}_{m}$​ at month $\:m$, and known observations $\:{y}_{m-1}$ and $\:{y}_{m+1}$ in the adjacent months. For a highly correlated reference lake, the corresponding observations are $\:{x}_{m-1\:}$, $\:{x}_{m}\:$and $\:{x}_{m+1}$​​. A proportional relationship between the two lakes is assumed:

$$\frac{{x_{{m - 1}} }}{{y_{{m - 1}} }} = \frac{{x_{m} }}{{y_{m} }} = \frac{{x_{{m + 1}} }}{{y_{{m + 1}} }}$$

This relationship implies consistent relative dynamics between the target and reference lakes across consecutive months. Based on this, the missing value $\:{y}_{m}$ can be estimated using the following expression:

$$y_{m} = \left\{ {\begin{array}{*{20}l} {\frac{{2x_{m} y_{{m + 1}} y_{{m - 1}} }}{{x_{{m + 1}} y_{{m - 1}} + x_{{m - 1}} y_{{m + 1}} }},~if~x_{{m - 1}} x_{m} x_{{m + 1}} y_{{m - 1}} y_{{m + 1}} \ne 0~~} \hfill \\ {~\frac{{x_{m} y_{{m - 1}} }}{{x_{{m - 1}} }},~~~~~~~~~~~~~~~~~~~~~~~~~if~x_{{m - 1}} x_{m} y_{{m - 1}} \ne 0} \hfill \\ {~\frac{{x_{m} y_{{m + 1}} }}{{x_{{m + 1}} }},~~~~~~~~~~~~~~~~~~~~~~~~~if~x_{m} x_{{m + 1}} y_{{m + 1}} \ne 0} \hfill \\ \end{array} } \right.$$

This piecewise estimation approach allows for flexibility based on data availability, while preserving proportional relationships between temporally aligned observations from correlated lakes.

While this approach captures key spatial signals, relying solely on a single reference lake introduces vulnerability to anomalies or unrepresentative fluctuations. To enhance robustness, the SNCI framework extends this strategy by incorporating multiple spatially correlated lakes, drawing methodological inspiration from Geographically Weighted Regression^31^. Each neighboring lake contributes to the estimation in proportion to its correlation strength with the target lake, thereby reducing the influence of outliers and improving resilience under varying hydrological conditions.

The final weighted interpolation strategy aggregates information from $\:n$ correlated neighboring lakes. The interpolated area $\:{y}_{m}$ is given by:

$$~y_{m} = \mathop \sum \limits_{{i = 1}}^{n} w_{i} \cdot x_{{m,i}} \quad {\text{where~}}\;w_{i} = \frac{{\left| {P\left( {x_{i} ,y} \right)} \right|}}{{\mathop \sum \nolimits_{{j = 1}}^{n} \left| {P\left( {x_{j} ,y} \right)} \right|}}~~$$

Here, $\:{x}_{m,i}$ denotes the observed area of the $\:i$-th neighboring lake at month $\:m$; $\:{w}_{i}$ indicates the weight assigned to each neighboring lake, computed based on the Pearson coefficient $\:P({x}_{i},y)$ between the lake area time series of the $\:i$-th neighboring lake and the target lake. Consequently, lakes exhibiting stronger correlations receive larger weights and thus exert proportionally greater influence on the interpolation results.

To ensure transparency and reproducibility, we explicitly define the weight assignment mechanism used in the SNCI framework. The above formulation normalizes the absolute correlation values such that all weights sum to 1. No additional transformations (e.g., squaring or clipping) are applied, preserving the original proportional relationships and interpretability.

In cases where no neighboring lakes exceed the predefined correlation threshold (0.4), the four most correlated lakes are selected as references, regardless of their coefficients. If fewer than four candidates exist, all available lakes are used. This fallback ensures SNCI applicability in isolated or weakly correlated regions.

### Baseline methods and evaluation metrics

To validate the effectiveness of the proposed SNCI method, we conducted a comparative analysis against four representative interpolation baselines commonly employed in spatiotemporal environmental modeling. These include: (1) Linear Interpolation: A simple method that estimates missing values by linearly connecting two adjacent valid observations. (2) Polynomial Fitting: Applies a second-order polynomial to each lake’s time series to infer gaps based on smooth temporal trends. (3) LSTM: A recurrent neural network architecture capable of capturing complex temporal dependencies, with a separate LSTM model trained for each lake using its historical observations. (4) Random Forest (RF): A non-parametric machine learning method that leverages time-lagged input features to predict missing values in the series.

The performance of these methods was validated using monthly lake area estimates derived from the DW dataset for the period 2017–2020. To construct monthly reference lake areas, we applied a temporal compositing approach: for each lake and month, all valid DW observations were collected. A pixel was marked as water if it was labeled as such in any observation during that month. Monthly lake extent was then computed by summing water pixels within the buffered lake boundary. This method reduces underestimation from cloud cover and acquisition gaps.

With the reference values derived, we adopted a leave-one-month-out evaluation strategy to simulate realistic missing data scenarios. Specifically, for each lake and each month during 2017–2020, the GSW-derived lake area was masked, interpolation was performed using the remaining time series, and the interpolated result was validated against the corresponding DW-derived ground truth. This ensured a systematic and temporally balanced evaluation across all methods.

This setup avoids the randomness associated with masked-value simulation and enables month-specific performance analysis, which is particularly useful for identifying seasonal sensitivities or method-specific weaknesses.

Traditional interpolation methods (e.g., linear and polynomial fitting) were directly applied to the incomplete GSW time series. In contrast, the LSTM and RF models were trained on each lake’s complete GSW records from 2000 to 2016 and evaluated on the masked gaps during 2017–2020. This consistent train-test protocol ensured fair temporal comparison across all methods.

To enhance reproducibility, key model parameters were standardized. For the LSTM model, we used a single-layer LSTM with 64 hidden units and a fully connected output layer. Models were trained for 200 epochs with a batch size of 16 using the Adam optimizer (learning rate = 0.001). A 12-month sliding window was used as input, with zero-padding for short sequences. For the RF model, we used an ensemble of 100 decision trees with a maximum depth of 8. The input consisted of the 12 preceding monthly lake areas (lagged features). All models were implemented using TensorFlow and Scikit-learn, with hyperparameters tuned via grid search.

Interpolation performance was evaluated using three types of metrics:

Mean Absolute Error (MAE) and Root Mean Square Error (RMSE) to quantify absolute and variance-weighted deviations:

$${\text{MAE}} = \frac{1}{n}\mathop \sum \limits_{{i = 1}}^{n} \left| {\hat{y}_{i} - y_{i} } \right|$$

$${\text{RMSE}} = \sqrt {\frac{1}{n}\mathop \sum \limits_{{i = 1}}^{n} \left( {\hat{y}_{i} - y_{i} } \right)^{2} }$$

Pearson Correlation Coefficient (P) to evaluate temporal consistency between interpolated and reference series:

$$P = \frac{{\mathop \sum \nolimits_{{i = 1}}^{n} \left( {\hat{y}_{i} - \overline{{\hat{y}}} } \right)\left( {y_{i} - \bar{y}} \right)}}{{\sqrt {\mathop \sum \nolimits_{{i = 1}}^{n} (\overline{{\hat{y}}} - \overline{{\hat{y}}} )^{2} } \cdot \sqrt {\mathop \sum \nolimits_{{i = 1}}^{n} (y_{i} - \bar{y})^{2} } }}$$

where $\:{y}_{i}$ and $\:{\overline{y}}_{i}$ represent the ground-truth and interpolated lake areas at time $\:i$, and $\:n$ is the number of missing points being evaluated.

All metrics were computed on a per-lake basis, and their regional averages were reported to reflect both local accuracy and overall robustness. This evaluation framework provides a reproducible and rigorous basis for comparing interpolation methods under varying levels of data sparsity.

## Results

### Lake area extraction and outlier removal

**Fig. 5:**
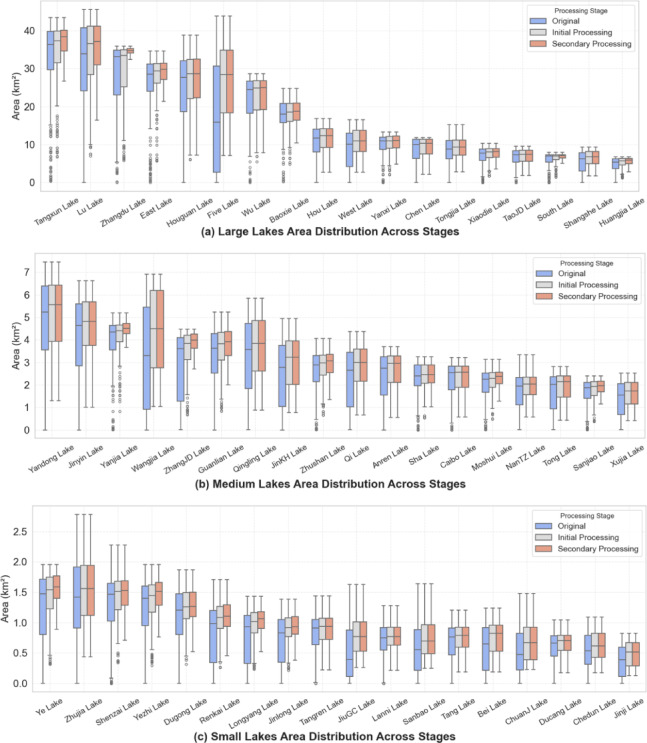
Distribution of lake surface area data before and after outlier removal. The figure shows lake area distributions under three conditions: (1) raw data, (2) after initial screening using the GSW permanent water dataset, and (3) after further filtering via Boxplot analysis.

To address the prevalence of anomalously low values—often caused by cloud cover, acquisition gaps, or sensor artifacts—a two-stage outlier removal strategy was implemented. As illustrated in Fig. 5, the first step employed the GSW permanent water dataset as a baseline reference, removing monthly lake area values substantially lower than historical minimum extents. This step effectively eliminated extreme outliers, as seen by the reduced dispersion in the middle boxplot. A second refinement step applied a 1.5×IQR boxplot-based filter to detect residual anomalies, particularly subtle deviations not captured by the initial screening, further improving data quality (rightmost boxplot).

This two-stage procedure removed an average of 10.0%, 12.3%, and 17.8% of samples from large, medium, and small lakes, respectively, during the initial filtering step. The secondary refinement further eliminated 15.9%, 16.0%, and 20.4% of samples within each category. Lakes with pronounced anomalies in the raw measurements—such as Five Lake and Wangjia Lake—underwent the most substantial reductions.

Importantly, the mean lake area values increased across all groups following both processing steps, indicating successful removal of low outliers. For instance, Five Lake exhibited a 54.8% increase in mean area (from 17.27 km² to 26.73 km²), while Renkai Lake and JiuGC Lake showed increases of 33.0% and 58.7%, respectively. These results confirm that the adopted cleaning strategy substantially mitigates the effects of noise and artifacts, thereby improving the reliability of lake area time series for subsequent interpolation analyses.

**Fig. 6:**
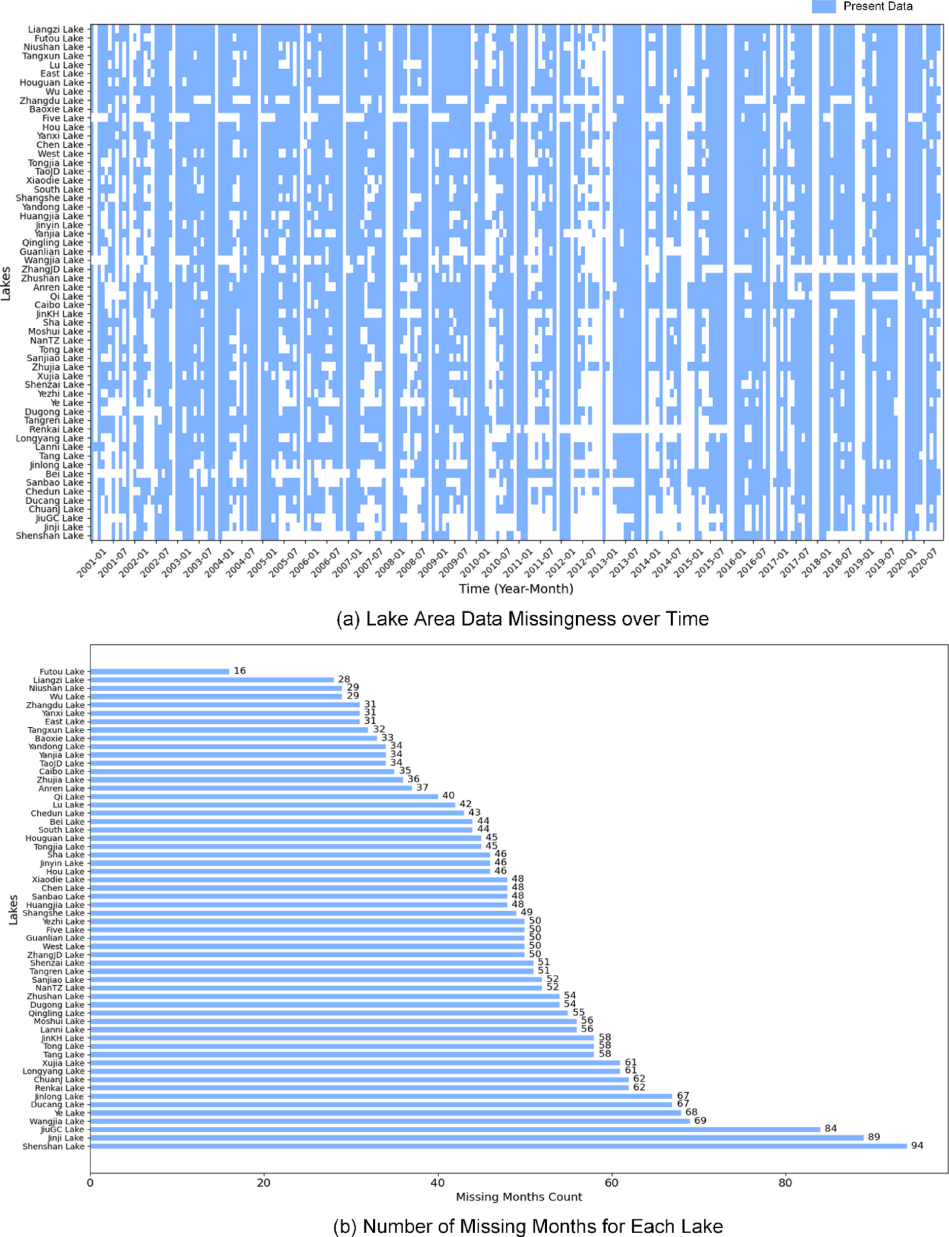
Summary of missing lake surface area records. (**a**) Time series completeness across all lakes, where blue bars indicate valid observations and white gaps denote missing values. (**b**) Histogram of missing months per lake, illustrating the spatial variability in data availability.

In Fig. 6a, blue bars denote months with valid observations, while white spaces indicate missing records. These gaps are not randomly scattered but rather tend to cluster around specific years—particularly from 2012 to 2014—implying the influence of persistent cloud cover or prolonged satellite acquisition gaps. Such temporal clustering violates the assumption of random missingness that underlies many traditional interpolation techniques.

Figure 6b further quantifies missing data across lakes, revealing that most exhibit between 20 and 60 months of missing records. A few lakes—such as Futuo, Nianhua, and Zhangjia—demonstrate relatively complete time series, likely due to favorable local observation conditions or morphological characteristics. On average, each lake exhibited 48.98 months of missing data, accounting for 20.41% of the full 240-month time series (2000–2020). This substantial proportion of missing observations underscores the need for robust interpolation techniques to ensure temporal continuity in long-term lake monitoring. In addition, the observed spatial variability highlights the importance of accounting for heterogeneity in both data quality and observation frequency when designing interpolation strategies.

### Spatial correlation results

**Fig. 7:**
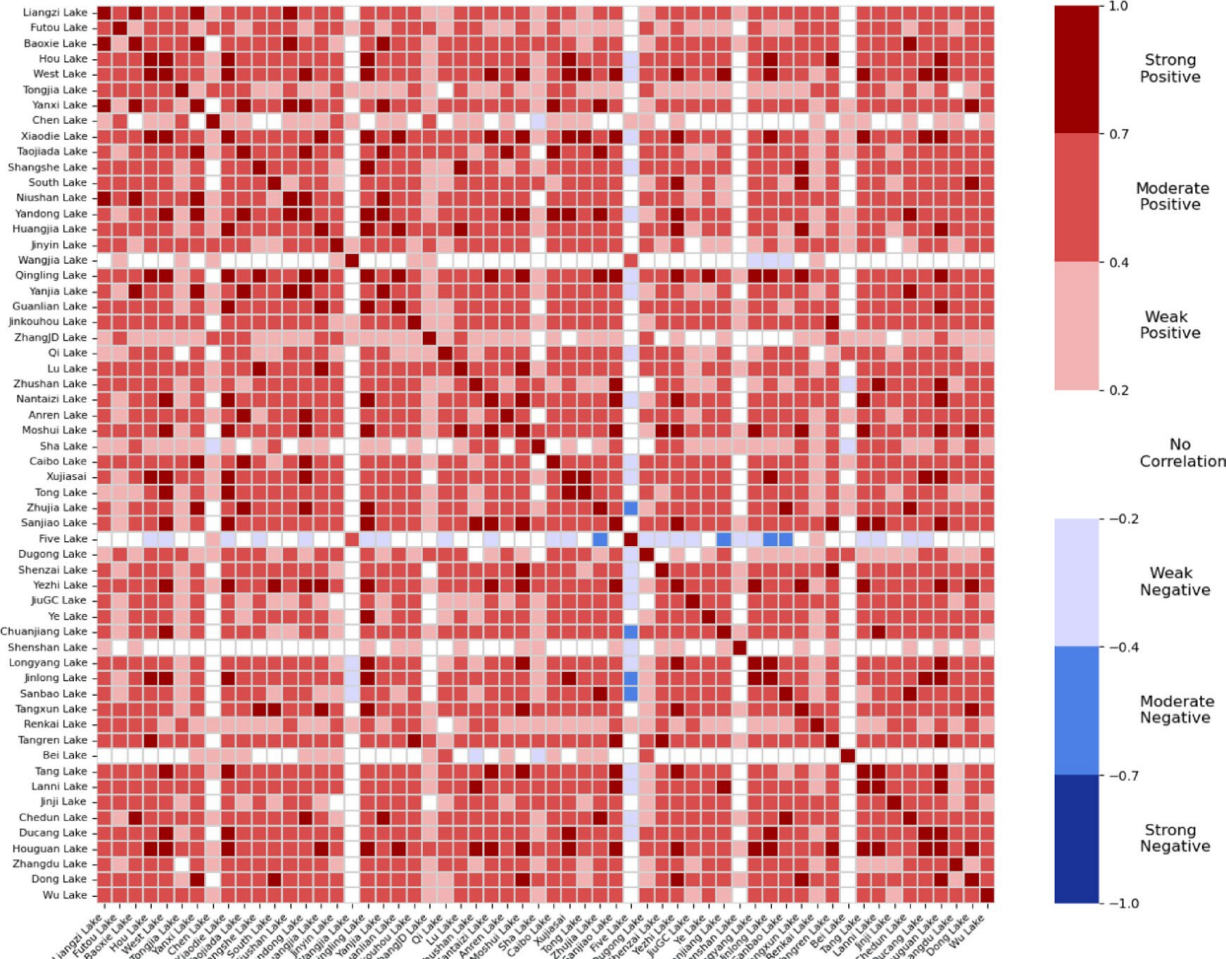
Correlation heatmap of lake area dynamics in the Wuhan region. Warmer colors indicate stronger positive correlations in temporal area changes between lake pairs, while cooler tones highlight weak or negative relationships.

Figure 7 illustrates that most lakes in the study area exhibit predominantly positive correlations, with only a few exceptions—such as Wangjia Lake and Bei Lake—showing weak or negligible correlation with others. Notably, Five Lake demonstrates a negative correlation with many other lakes, making it an additional outlier of particular interest. Although some lakes generally maintain low correlation levels with their neighbors, several lake pairs exhibit consistently high correlations, particularly when examining their temporal area changes. High correlation implies that these lakes experience synchronous expansions and contractions over time. That is, increases or decreases in lake area tend to occur concurrently across the pair. Even during abrupt changes, such as rapid growth or shrinkage, the timing of the variations remains aligned.

**Fig. 8:**
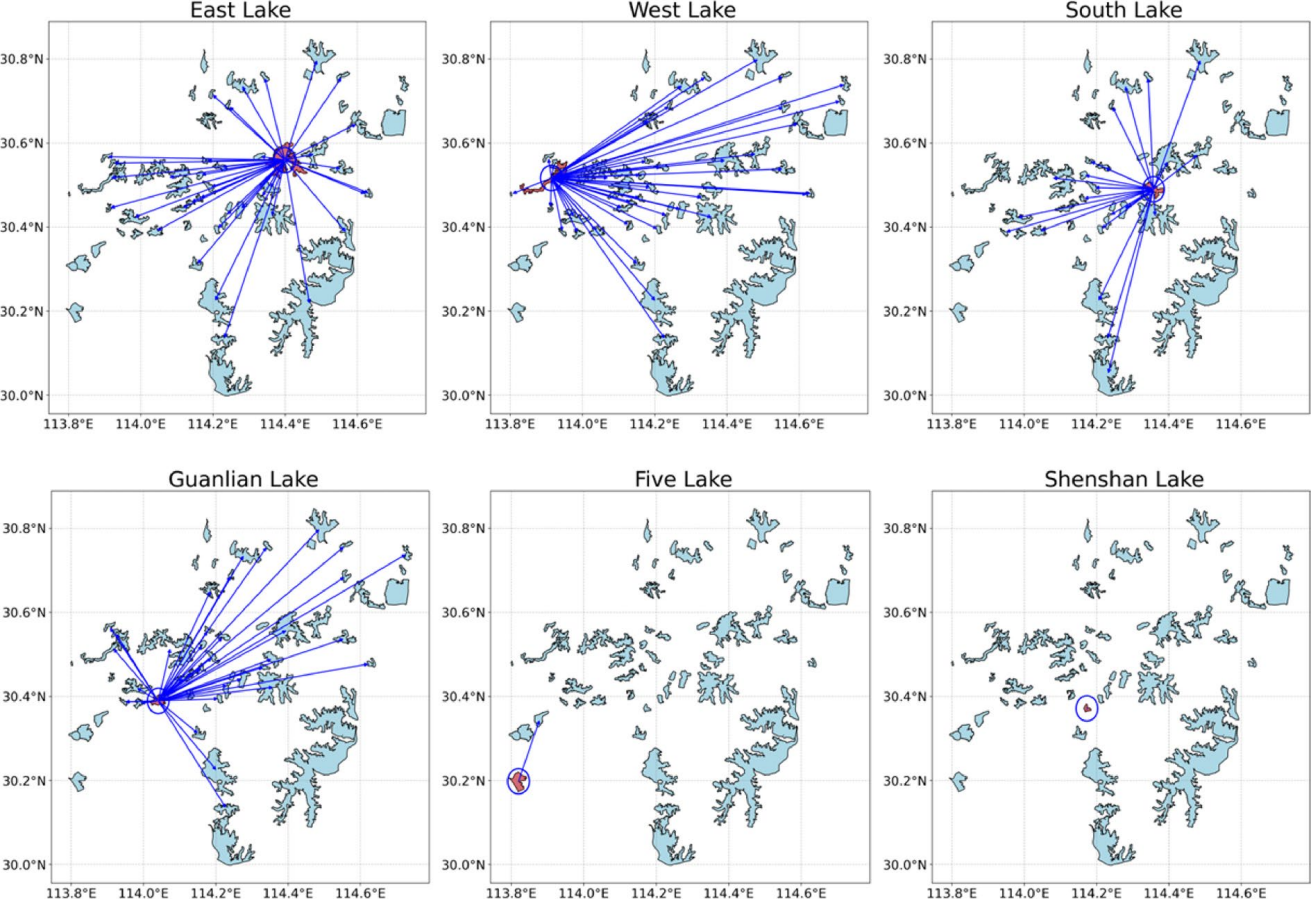
Highly correlated lake pairs centered on selected study lakes. The figure highlights neighboring lakes that exhibit strong temporal correlation in surface area change with several representative lakes, serving as potential candidates for SNCI-based interpolation.

To further reveal spatial structures in inter-lake correlations, Fig. 8 maps the correlation networks of six representative lakes. Blue lines connect lake pairs with Pearson coefficients above 0.4. A clear spatial clustering pattern emerges: lakes such as East Lake and South Lake, located in central urban areas, show high correlation with numerous nearby lakes—consistent with Tobler’s First Law of Geography. In contrast, lakes like Shenshan and Five Lake exhibit fewer high-correlation links, likely due to hydrological isolation or local disturbances. These patterns confirm the presence of spatial heterogeneity, aligning with the Law of Spatial Heterogeneity, and imply that interpolation models like SNCI must be locally adaptive rather than globally uniform.

### Quantitative analysis of interpolation performance

A comprehensive quantitative analysis across all 54 lakes in the Wuhan region demonstrates that the SNCI method outperforms other interpolation approaches for most lakes, exhibiting robustness and generalizability. Compared with the second-best baseline (RF), SNCI reduces MAE and RMSE by an average of 18.6% and 9.9%, respectively, and achieves a median Pearson correlation coefficient of approximately 0.91. These aggregate results, detailed in Supplementary Table S1, validate SNCI’s applicability across diverse hydrological settings. A more detailed comparison is provided in Table 3 for the four representative lakes introduced in Sect. 2.5.

**Table 3:** Quantitative evaluation of different methods on four lakes.

Lake name	Metric	Linear	Polynomial	LSTM	RF	SNCI
East Lake	MAE ↓	0.840	0.632	0.452	0.505	0.252 (+ 50.1%)
	RMSE↓	1.289	0.937	0.784	0.671	0.481 (+ 28.3%)
	Correlation↑	0.759	0.880	0.918	0.947	0.971 (+ 2.5%)
South Lake	MAE↓	0.129	0.107	0.082	0.086	0.080 (+ 7.0%)
	RMSE↓	0.173	0.141	0.119	0.163	0.101 (+ 38.0%)
	Correlation↑	0.783	0.859	0.902	0.879	0.948 (+ 7.8%)
West Lake	MAE↓	2.080	1.564	1.329	0.972	0.549 (+ 43.5%)
	RMSE↓	2.858	2.074	1.821	1.279	1.131 (+ 11.6%)
	Correlation↑	0.604	0.811	0.858	0.950	0.956 (+ 0.6%)
Five Lake	MAE↓	5.754	3.571	2.940	2.865	2.899 (− 1.2%)
	RMSE↓	8.027	5.054	6.798	4.459	3.994 (+ 10.4%)
	Correlation↑	0.661	0.879	0.786	0.907	0.945 (+ 4.2%)

↓ indicates that lower values are better; ↑ indicates that higher values are better. Underlined values represent the best performance for each metric. Random Forest (RF) is used as the benchmark method, and the values in parentheses indicate the relative improvement of the proposed SNCI method compared to RF.

Across the board, SNCI delivers the lowest MAE and RMSE and highest correlation coefficients in nearly all cases. For instance, in East Lake, SNCI reduces MAE by 50.1% and RMSE by 28.3% compared to the strongest baseline (RF), showcasing its advantage in stable, well-regulated urban lakes. In West Lake, which exhibits more variability, SNCI still achieves a 43.5% MAE reduction and an 11.6% RMSE reduction. Even in the highly dynamic Five Lake, where RF slightly outperforms SNCI in MAE, SNCI attains better RMSE and correlation, indicating greater robustness and temporal fidelity.

**Fig. 9:**
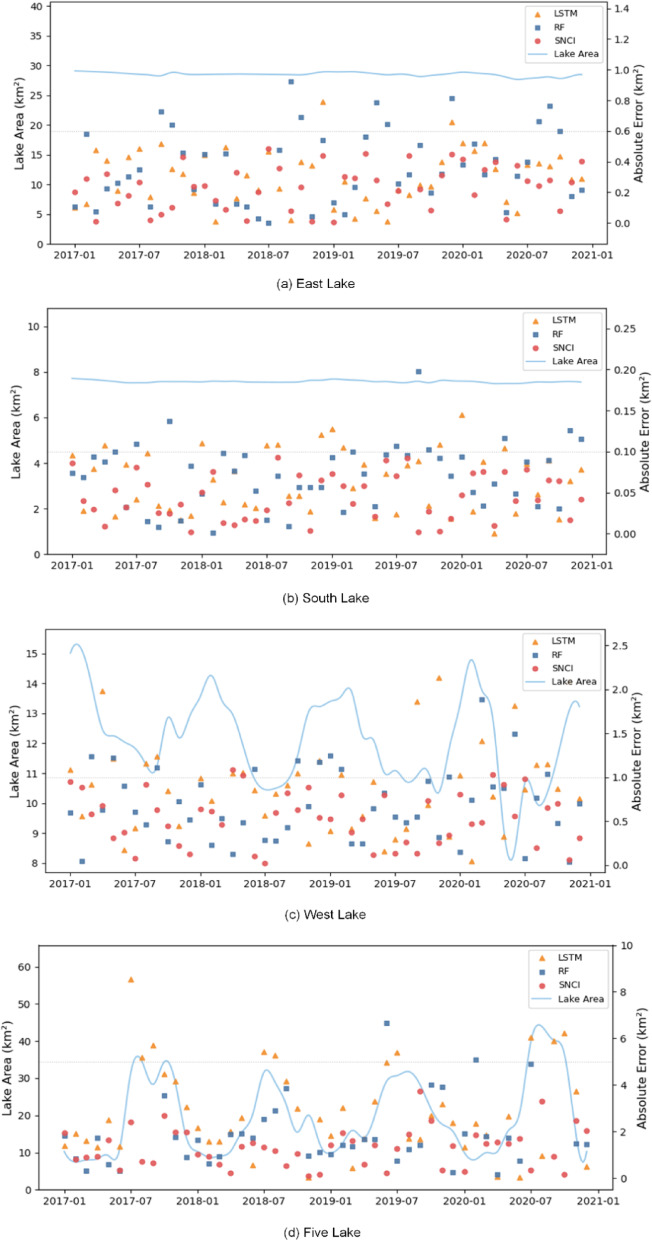
Comparison of reconstructed monthly lake areas from 2017 to 2020 using three selected methods: RF, LSTM, and the proposed SNCI. These methods were chosen for visualization due to their superior numerical performance.

To provide a clearer comparison across different lakes and models, Fig. 9 illustrates the reconstructed monthly lake areas from 2017 to 2020 for four representative lakes using three top-performing methods: RF, LSTM, and the proposed SNCI. In each subplot, the blue curve represents the observed lake area (left axis), while the absolute errors for each method are shown as scatter points (right axis), with orange triangles for LSTM, blue squares for RF, and red circles for SNCI.

In East Lake and South Lake (Fig. 9a and b), where seasonal variations are relatively moderate due to effective hydrological regulation and urban water management, all three methods demonstrate a reasonable ability to follow the temporal trends. However, SNCI achieves consistently lower errors, particularly during periods of sparse observations, owing to its capacity to exploit inter-lake spatial correlations. In contrast, West Lake (Fig. 9c), which lies in an urban–rural transition zone and exhibits stronger interannual variability, poses greater challenges for traditional models. LSTM and RF struggle to capture abrupt transitions and extreme events, often exceeding the 1 km² error threshold. SNCI, by contrast, demonstrates improved robustness, maintaining low errors while accurately capturing both the timing and amplitude of lake area changes. The challenge is even more pronounced in Five Lake (Fig. 9d), which exhibits substantial seasonal and interannual fluctuations, with areas ranging from 10 to over 50 km². Here, LSTM and RF tend to underestimate peak values and suffer from error instability, while SNCI is able to closely follow the observed dynamics with reduced error magnitude across the entire period.

These results further support the conclusion that SNCI not only achieves superior quantitative performance, but also provides more reliable and interpretable reconstructions under diverse lake conditions.

### Case studies of representative lakes

As introduced in Sect. 2.5, four representative lakes—East Lake, South Lake, West Lake, and Five Lake—were selected to reflect a range of hydrological settings, lake sizes, data availability, and degrees of anthropogenic influence. Building on the quantitative evaluation across all 54 lakes, this section presents a case study to qualitatively illustrate how the SNCI method can be applied to reconstruct lake area dynamics under varying seasonal and interannual conditions.

**Fig. 10:**
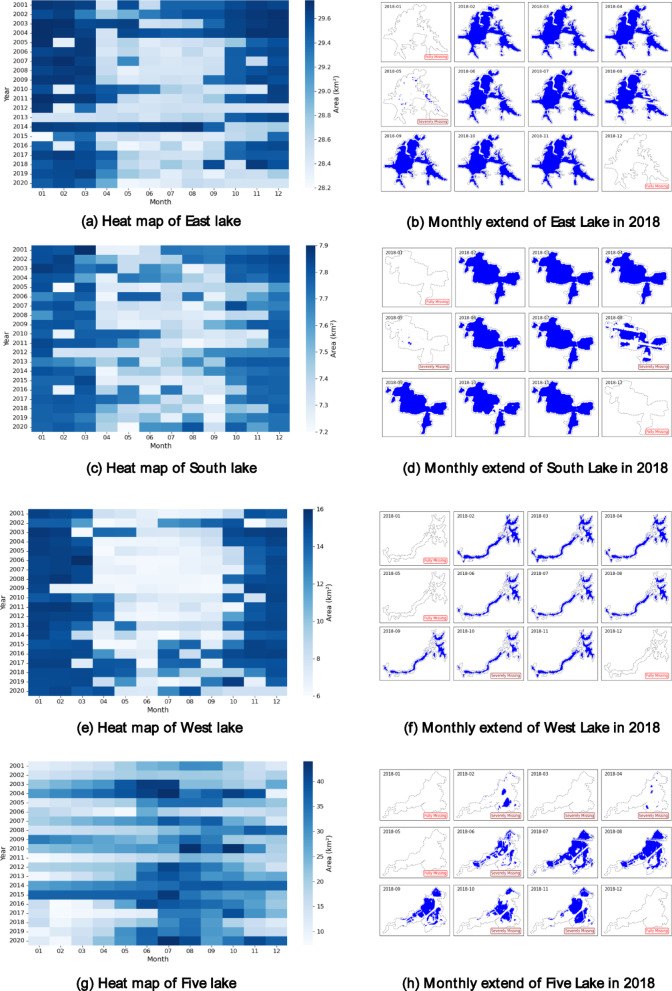
presents heatmap-based reconstructions of monthly lake area from 2000 to 2020 (left column), alongside the original, un-interpolated GSW-derived lake extents for the year 2018 (right column). The 2018 panels clearly reveal persistent data gaps—especially in winter months—caused by cloud cover and limited satellite observations, underscoring the need for robust interpolation techniques.

Figure 10 Monthly lake area dynamics from 2000 to 2020 for four representative lakes. The left column displays SNCI-interpolated lake areas as heatmaps, where missing values have been reconstructed using the proposed method. The right column shows the original GSW-derived lake extents for the year 2018, included to illustrate spatial morphology and seasonal variation under real observation conditions. These right-side panels are not interpolated and may contain data gaps due to cloud cover or satellite acquisition limitations.

Across the four lakes, the SNCI method produces temporally coherent area estimates. For urban lakes such as East Lake and South Lake, seasonal consistency and low-amplitude summer drawdowns are maintained, likely reflecting managed hydrological conditions. For suburban and rural lakes like West Lake and Five Lake, more dynamic seasonal and interannual variations are captured, suggesting that SNCI is well-suited to less regulated systems. These patterns demonstrate that SNCI adapts effectively to both stable and variable lake environments.

Although individual lake behavior is shaped by localized hydrometeorological and anthropogenic factors, the consistent spatial–temporal coherence observed across all cases suggests that SNCI offers a robust and generalizable interpolation strategy. Further interpretation of these patterns and their implications is provided in the Discussion section.

## Discussion

The SNCI interpolation method offers clear advantages for reconstructing lake area time series by leveraging spatial coherence among adjacent water bodies. Compared to conventional interpolation techniques and machine learning models, SNCI consistently achieves higher accuracy. These improvements underscore the value of incorporating spatial semantic information into hydrological monitoring, especially when addressing temporal discontinuities. Across all 54 lakes in the Wuhan region, SNCI reduced MAE and RMSE by 18.6% and 9.9%, respectively, relative to the best-performing baseline (RF). In well-regulated urban lakes, such as East Lake, gains were even more substantial—with over 50% reduction in MAE and nearly 30% in RMSE—highlighting SNCI’s strong generalizability across varying lake conditions.

This performance pattern is exemplified by the four representative lakes. In East Lake and South Lake, where hydrological conditions are relatively stable and shaped by urban water regulation, SNCI preserves seasonal consistency and smooth interannual trends. In contrast, West Lake—situated at the urban–rural interface—and Five Lake, which exhibits greater seasonal and interannual fluctuations, demonstrate SNCI’s robustness in capturing complex dynamics and abrupt changes. These qualitative results further validate SNCI’s adaptability across diverse lake environments and reinforce its utility under both regulated and dynamic hydrological scenarios.

The method’s effectiveness, however, is closely tied to the spatial distribution and hydrological connectivity of lakes in a given region. Our results from the Wuhan area—characterized by dense lake networks and strong hydrological links—demonstrate high reconstruction accuracy, attributable to stable inter-lake correlations. In contrast, applications in regions such as Beijing-Tianjin-Hebei revealed limited improvement, primarily due to sparse lake distributions and human interventions like the South-to-North Water Diversion Project^47^. Similarly, the Tibetan Plateau poses unique challenges due to complex climatic conditions^48,49^, persistent cloud cover, and resulting gaps in optical observations, all of which reduce inter-lake correlation strength and limit SNCI’s effectiveness^50^. These findings underscore the necessity of evaluating spatial correlation and data availability before applying SNCI in new settings.

Furthermore, the statistical foundation of SNCI brings both strengths and limitations. The current use of Pearson correlation assumes a linear and synchronous relationship between lake area time series. While appropriate in many hydrologically connected systems, this assumption may not capture nonlinear dependencies or time-lagged effects introduced by groundwater recharge, regulated flows, or meteorological events. Although Pearson correlation was selected for its simplicity and computational efficiency—essential for large-scale unsupervised applications—future work should explore alternative similarity metrics such as mutual information^51^, dynamic time warping^52^, or nonlinear correlation indices^53^. These alternatives may better capture complex hydrological dynamics, especially in regions with asynchronous or nonlinear interactions.

In addition, the current neighborhood construction in SNCI is based purely on statistical similarity, without incorporating geographic distance, climatic zones, lake morphology, watershed affiliation, or anthropogenic influences. This may limit its physical interpretability and predictive accuracy, particularly in hydrologically diverse or human-impacted regions. Integrating geospatial constraints or auxiliary environmental variables—such as precipitation, evapotranspiration, or lake typology—could enhance the robustness and generalizability of SNCI, especially in heterogeneous landscapes^54^. Expanding the spatial neighborhood search radius may also improve adaptability in regions with sparsely distributed lakes.

From a methodological perspective, SNCI offers a favorable balance between simplicity, interpretability, and performance. Compared with geographically weighted regression^31^, which prioritizes proximity over semantic consistency, or graph neural networks^25^, which require substantial labeled data and computational resources, SNCI is more accessible and computationally efficient. Its correlation-weighted multi-neighbor scheme stabilizes interpolated values and enhances robustness, especially under frequent observation loss. This makes SNCI particularly well-suited for large-scale and long-term lake monitoring across diverse hydrological and environmental conditions.

Finally, while SNCI currently focuses on spatial interpolation, future extensions could incorporate temporal forecasting capabilities. Hybrid models that integrate SNCI with temporal prediction techniques—such as LSTM or Temporal Convolutional Networks^55^—could further improve performance under persistent observation gaps or seasonal anomalies. By explicitly combining spatial semantics with temporal dynamics, such integrated approaches could enhance both accuracy and adaptability. Overall, although SNCI already demonstrates strong practical utility, particularly in cloud-prone or data-limited regions, future work should explore hybrid frameworks, nonlinear correlation metrics, and geospatial context integration to fully realize its potential.

## Conclusion

Accurate lake area estimation is essential for hydrological modeling, environmental surveillance, and sustainable water resource management. However, optical satellite observations frequently suffer from temporal discontinuities due to persistent cloud cover and sensor limitations. To address these challenges, this study introduces a novel interpolation framework—Semantic Neighborhood Correlation—that reconstructs missing lake area values by exploiting spatiotemporal correlations among neighboring lakes.

The proposed SNCI method offers a distinct advantage over conventional interpolation approaches by incorporating spatial semantics, thereby enabling more accurate reconstruction under conditions of seasonal and interannual variability. Experimental evaluations using the GSW dataset, validated against the high-resolution DW dataset, demonstrate that SNCI consistently outperforms traditional techniques such as linear interpolation, polynomial fitting, and random forest models. Notably, in representative lakes such as East Lake (see Table 3), SNCI achieves up to a 50.1% reduction in MAE and a 28.3% reduction in RMSE compared to the strongest baseline (RF). Moreover, across all 54 lakes (see Supplementary Table S1), SNCI maintains superior overall performance in terms of MAE, RMSE, and correlation, confirming its robustness and generalizability.

In conclusion, SNCI provides a scalable, interpretable, and computationally efficient solution for reconstructing missing lake area observations. Its ability to jointly capture spatial coherence and temporal stability renders it particularly valuable for long-term, large-scale monitoring of inland surface water dynamics. As satellite observation archives continue to grow and global water studies demand increasingly precise data, SNCI holds strong potential to support the next generation of remote sensing–based hydrological assessments.

## Electronic supplementary material

Below is the link to the electronic supplementary material.

Supplementary Material 1.

## Data Availability

In our experimental data, all datasets are publicly available. The GSW (Global Surface Water) dataset and DW (Dynamic Water) dataset are openly accessible via Google Earth Engine (GEE). The China-LDRL dataset is obtained from the study by Wang et al. (2022) titled “Improved Maps of Surface Water Bodies, Large Dams, Reservoirs, and Lakes in China”, published in Earth System Science Data (DOI: 10.5194/essd-14-3757-2022).Additional supplementary materials, including implementation codes, are publicly available at our GitHub repository: https://github.com/Tian8du/SSC-Codes. For any inquiries regarding data access, please contact liu_chen@whu.edu.cn.
