# Supplementary material for: Spatially-informed interpolation for reconstructing lake area time series using semantic neighborhood correlation
Source: Sci Rep. 2025 Jul 9;15:24787. doi: 10.1038/s41598-025-09410-3 (PMC12241530; doi:10.1038/s41598-025-09410-3)
Supplement: Supplementary file 1 — Supplementary Material 1. [file 41598_2025_9410_MOESM1_ESM.docx]

Supplementary Table S1. Quantitative performance comparison of five interpolation methods across the remaining 50 lakes in the Wuhan region

| **Lake Name** | **Metric** | **Linear** | **Polynomial** | **LSTM** | **RF** | **SNCI** |
| --- | --- | --- | --- | --- | --- | --- |
| Liangzi Lake | MAE ↓ | 5.20 | 3.76 | 2.51 | 1.05 | 0.93 |
|  | RMSE ↓ | 6.23 | 4.48 | 2.93 | 1.36 | 1.61 |
|  | Correlation ↑ | 0.49 | 0.84 | 0.87 | 0.95 | 0.92 |
| Futou Lake | MAE ↓ | 2.32 | 1.79 | 0.86 | 0.33 | 0.48 |
|  | RMSE ↓ | 2.84 | 1.98 | 1.12 | 0.47 | 0.40 |
|  | Correlation ↑ | 0.51 | 0.79 | 0.88 | 0.95 | 0.91 |
| Niushan Lake | MAE ↓ | 1.37 | 0.68 | 0.39 | 0.09 | 0.46 |
|  | RMSE ↓ | 1.66 | 1.30 | 0.73 | 0.57 | 0.53 |
|  | Correlation ↑ | 0.59 | 0.82 | 0.88 | 0.94 | 0.91 |
| Tangxun Lake | MAE ↓ | 0.68 | 0.86 | 0.64 | 0.21 | 0.11 |
|  | RMSE ↓ | 1.30 | 1.03 | 0.15 | 0.48 | 0.25 |
|  | Correlation ↑ | 0.66 | 0.84 | 0.89 | 0.84 | 0.94 |
| Lu Lake | MAE ↓ | 1.03 | 0.82 | 0.42 | 0.21 | 0.19 |
|  | RMSE ↓ | 1.04 | 0.98 | 0.52 | 0.46 | 0.23 |
|  | Correlation ↑ | 0.78 | 0.81 | 0.89 | 0.91 | 0.90 |
| Houguan Lake | MAE ↓ | 0.84 | 0.48 | 0.32 | 0.16 | 0.34 |
|  | RMSE ↓ | 0.99 | 0.59 | 0.40 | 0.20 | 0.38 |
|  | Correlation ↑ | 0.61 | 0.83 | 0.88 | 0.94 | 0.94 |
| Wu Lake | MAE ↓ | 0.74 | 0.20 | 0.17 | 0.33 | 0.18 |
|  | RMSE ↓ | 0.67 | 0.30 | 0.54 | 0.37 | 0.15 |
|  | Correlation ↑ | 0.50 | 0.83 | 0.88 | 0.92 | 0.98 |
| Zhangdu Lake | MAE ↓ | 0.62 | 0.81 | 0.61 | 0.30 | 0.18 |
|  | RMSE ↓ | 1.02 | 0.57 | 0.71 | 0.26 | 0.13 |
|  | Correlation ↑ | 0.49 | 0.81 | 0.88 | 0.90 | 0.91 |
| Baoxie Lake | MAE ↓ | 0.24 | 0.33 | 0.42 | 0.31 | 0.30 |
|  | RMSE ↓ | 0.75 | 0.21 | 0.47 | 0.16 | 0.12 |
|  | Correlation ↑ | 0.79 | 0.82 | 0.86 | 0.91 | 0.94 |
| Hou Lake | MAE ↓ | 0.17 | 0.21 | 0.06 | 0.07 | 0.06 |
|  | RMSE ↓ | 0.14 | 0.26 | 0.17 | 0.11 | 0.08 |
|  | Correlation ↑ | 0.56 | 0.81 | 0.89 | 0.84 | 0.87 |
| Yanxi Lake | MAE ↓ | 0.45 | 0.19 | 0.13 | 0.26 | 0.26 |
|  | RMSE ↓ | 0.11 | 0.13 | 0.05 | 0.28 | 0.13 |
|  | Correlation ↑ | 0.54 | 0.78 | 0.88 | 0.92 | 0.91 |
| Chen Lake | MAE ↓ | 0.22 | 0.16 | 0.31 | 0.15 | 0.05 |
|  | RMSE ↓ | 0.27 | 0.40 | 0.06 | 0.27 | 0.06 |
|  | Correlation ↑ | 0.68 | 0.84 | 0.86 | 0.92 | 0.89 |
| Tongjia Lake | MAE ↓ | 0.42 | 0.16 | 0.11 | 0.09 | 0.05 |
|  | RMSE ↓ | 0.07 | 0.20 | 0.13 | 0.13 | 0.26 |
|  | Correlation ↑ | 0.63 | 0.83 | 0.88 | 0.93 | 0.89 |
| TaoJD Lake | MAE ↓ | 0.36 | 0.12 | 0.08 | 0.16 | 0.04 |
|  | RMSE ↓ | 0.40 | 0.15 | 0.30 | 0.35 | 0.25 |
|  | Correlation ↑ | 0.79 | 0.77 | 0.89 | 0.90 | 0.90 |
| Xiaodie Lake | MAE ↓ | 0.19 | 0.06 | 0.29 | 0.25 | 0.16 |
|  | RMSE ↓ | 0.23 | 0.37 | 0.32 | 0.16 | 0.25 |
|  | Correlation ↑ | 0.60 | 0.81 | 0.90 | 0.91 | 0.89 |
| Shangshe Lake | MAE ↓ | 0.05 | 0.31 | 0.13 | 0.16 | 0.17 |
|  | RMSE ↓ | 0.39 | 0.14 | 0.29 | 0.05 | 0.04 |
|  | Correlation ↑ | 0.64 | 0.78 | 0.89 | 0.92 | 0.90 |
| Yandong Lake | MAE ↓ | 0.08 | 0.09 | 0.26 | 0.05 | 0.03 |
|  | RMSE ↓ | 0.15 | 0.09 | 0.28 | 0.14 | 0.09 |
|  | Correlation ↑ | 0.57 | 0.83 | 0.86 | 0.90 | 0. 92 |
| Huangjia Lake | MAE ↓ | 0.33 | 0.30 | 0.27 | 0.23 | 0.23 |
|  | RMSE ↓ | 0.16 | 0.12 | 0.08 | 0.24 | 0.16 |
|  | Correlation ↑ | 0.65 | 0.83 | 0.87 | 0.83 | 0.91 |
| Jinyin Lake | MAE ↓ | 0.29 | 0.28 | 0.25 | 0.23 | 0.22 |
|  | RMSE ↓ | 0.23 | 0.30 | 0.23 | 0.27 | 0.23 |
|  | Correlation ↑ | 0.68 | 0.82 | 0.87 | 0.82 | 0.88 |
| Yanjia Lake | MAE ↓ | 0.11 | 0.12 | 0.15 | 0.17 | 0.18 |
|  | RMSE ↓ | 0.33 | 0.10 | 0.13 | 0.23 | 0.23 |
|  | Correlation ↑ | 0.79 | 0.78 | 0.89 | 0.90 | 0.88 |
| Qingling Lake | MAE ↓ | 0.21 | 0.17 | 0.24 | 0.22 | 0.12 |
|  | RMSE ↓ | 0.21 | 0.28 | 0.26 | 0.23 | 0.23 |
|  | Correlation ↑ | 0.61 | 0.82 | 0.89 | 0.94 | 0.89 |
| Guanlian Lake | MAE ↓ | 0.29 | 0.27 | 0.16 | 0.18 | 0.22 |
|  | RMSE ↓ | 0.31 | 0.29 | 0.06 | 0.09 | 0.17 |
|  | Correlation ↑ | 0.76 | 0.79 | 0.88 | 0.94 | 0.89 |
| Wangjia Lake | MAE ↓ | 0.30 | 0.28 | 0.15 | 0.23 | 0.09 |
|  | RMSE ↓ | 0.33 | 0.20 | 0.27 | 0.23 | 0.13 |
|  | Correlation ↑ | 0.77 | 0.77 | 0.88 | 0.92 | 0.92 |
| ZhangJD Lake | MAE ↓ | 0.29 | 0.27 | 0.25 | 0.22 | 0.18 |
|  | RMSE ↓ | 0.28 | 0.21 | 0.26 | 0.23 | 0.23 |
|  | Correlation ↑ | 0.74 | 0.84 | 0.89 | 0.95 | 0.89 |
| Zhushan Lake | MAE ↓ | 0.27 | 0.05 | 0.17 | 0.12 | 0.19 |
|  | RMSE ↓ | 0.21 | 0.27 | 0.14 | 0.12 | 0.18 |
|  | Correlation ↑ | 0.80 | 0.80 | 0.87 | 0.92 | 0.89 |
| Anren Lake | MAE ↓ | 0.26 | 0.25 | 0.27 | 0.22 | 0.19 |
|  | RMSE ↓ | 0.22 | 0.26 | 0.24 | 0.18 | 0.18 |
|  | Correlation ↑ | 0.55 | 0.80 | 0.87 | 0.91 | 0.92 |
| Qi Lake | MAE ↓ | 0.27 | 0.15 | 0.17 | 0.22 | 0.12 |
|  | RMSE ↓ | 0.12 | 0.06 | 0.14 | 0.22 | 0.09 |
|  | Correlation ↑ | 0.73 | 0.84 | 0.86 | 0.91 | 0.93 |
| Caibo Lake | MAE ↓ | 0.26 | 0.04 | 0.03 | 0.07 | 0.05 |
|  | RMSE ↓ | 0.13 | 0.25 | 0.17 | 0.18 | 0.12 |
|  | Correlation ↑ | 0.71 | 0.81 | 0.86 | 0.93 | 0.91 |
| JinKH Lake | MAE ↓ | 0.13 | 0.25 | 0.16 | 0.12 | 0.12 |
|  | RMSE ↓ | 0.29 | 0.27 | 0.25 | 0.21 | 0.22 |
|  | Correlation ↑ | 0.78 | 0.81 | 0.90 | 0.90 | 0.91 |
| Sha Lake | MAE ↓ | 0.14 | 0.16 | 0.17 | 0.19 | 0.09 |
|  | RMSE ↓ | 0.13 | 0.15 | 0.16 | 0.22 | 0.12 |
|  | Correlation ↑ | 0.71 | 0.77 | 0.90 | 0.93 | 0.90 |
| Moshui Lake | MAE ↓ | 0.15 | 0.16 | 0.23 | 0.19 | 0.09 |
|  | RMSE ↓ | 0.23 | 0.25 | 0.13 | 0.22 | 0.07 |
|  | Correlation ↑ | 0.72 | 0.83 | 0.86 | 0.91 | 0.98 |
| NanTZ Lake | MAE ↓ | 0.15 | 0.24 | 0.22 | 0.19 | 0.19 |
|  | RMSE ↓ | 0.16 | 0.16 | 0.17 | 0.19 | 0.19 |
|  | Correlation ↑ | 0.56 | 0.77 | 0.87 | 0.91 | 0.89 |
| Tong Lake | MAE ↓ | 0.25 | 0.33 | 0.18 | 0.19 | 0.11 |
|  | RMSE ↓ | 0.26 | 0.16 | 0.23 | 0.19 | 0.09 |
|  | Correlation ↑ | 0.51 | 0.83 | 0.86 | 0.84 | 0.94 |
| Sanjiao Lake | MAE ↓ | 0.25 | 0.17 | 0.22 | 0.19 | 0.11 |
|  | RMSE ↓ | 0.26 | 0.24 | 0.23 | 0.19 | 0.19 |
|  | Correlation ↑ | 0.72 | 0.77 | 0.89 | 0.92 | 0.94 |
| Zhujia Lake | MAE ↓ | 0.16 | 0.23 | 0.22 | 0.19 | 0.11 |
|  | RMSE ↓ | 0.25 | 0.33 | 0.22 | 0.21 | 0.19 |
|  | Correlation ↑ | 0.59 | 0.80 | 0.86 | 0.92 | 0.92 |
| Xujia Lake | MAE ↓ | 0.36 | 0.23 | 0.18 | 0.21 | 0.11 |
|  | RMSE ↓ | 0.25 | 0.24 | 0.18 | 0.31 | 0.11 |
|  | Correlation ↑ | 0.75 | 0.77 | 0.89 | 0.91 | 0.93 |
| Shenzai Lake | MAE ↓ | 0.26 | 0.23 | 0.22 | 0.21 | 0.11 |
|  | RMSE ↓ | 0.35 | 0.27 | 0.18 | 0.19 | 0.17 |
|  | Correlation ↑ | 0.58 | 0.79 | 0.86 | 0.92 | 0.92 |
| Yezhi Lake | MAE ↓ | 0.24 | 0.23 | 0.18 | 0.21 | 0.21 |
|  | RMSE ↓ | 0.24 | 0.23 | 0.22 | 0.21 | 0.19 |
|  | Correlation ↑ | 0.70 | 0.80 | 0.87 | 0.93 | 0.89 |
| Ye Lake | MAE ↓ | 0.24 | 0.33 | 0.22 | 0.21 | 0.19 |
|  | RMSE ↓ | 0.25 | 0.27 | 0.22 | 0.32 | 0.21 |
|  | Correlation ↑ | 0.59 | 0.82 | 0.88 | 0.92 | 0.90 |
| Dugong Lake | MAE ↓ | 0.33 | 0.28 | 0.19 | 0.21 | 0.19 |
|  | RMSE ↓ | 0.34 | 0.23 | 0.22 | 0.18 | 0.12 |
|  | Correlation ↑ | 0.63 | 0.79 | 0.88 | 0.91 | 0.94 |
| Tangren Lake | MAE ↓ | 0.32 | 0.18 | 0.21 | 0.21 | 0.20 |
|  | RMSE ↓ | 0.23 | 0.22 | 0.19 | 0.21 | 0.21 |
|  | Correlation ↑ | 0.49 | 0.78 | 0.90 | 0.83 | 0.87 |
| Renkai Lake | MAE ↓ | 0.23 | 0.12 | 0.11 | 0.21 | 0.19 |
|  | RMSE ↓ | 0.33 | 0.18 | 0.22 | 0.19 | 0.21 |
|  | Correlation ↑ | 0.68 | 0.84 | 0.86 | 0.89 | 0.88 |
| Longyang Lake | MAE ↓ | 0.18 | 0.18 | 0.24 | 0.21 | 0.17 |
|  | RMSE ↓ | 0.23 | 0.22 | 0.22 | 0.23 | 0.19 |
|  | Correlation ↑ | 0.68 | 0.78 | 0.86 | 0.81 | 0.92 |
| Lanni Lake | MAE ↓ | 0.18 | 0.21 | 0.19 | 0.09 | 0.20 |
|  | RMSE ↓ | 0.18 | 0.23 | 0.19 | 0.19 | 0.14 |
|  | Correlation ↑ | 0.59 | 0.78 | 0.86 | 0.93 | 0.88 |
| Tang Lake | MAE ↓ | 0.18 | 0.19 | 0.21 | 0.17 | 0.14 |
|  | RMSE ↓ | 0.22 | 0.18 | 0.21 | 0.29 | 0.12 |
|  | Correlation ↑ | 0.67 | 0.83 | 0.90 | 0.91 | 0.92 |
| Jinlong Lake | MAE ↓ | 0.18 | 0.18 | 0.21 | 0.21 | 0.12 |
|  | RMSE ↓ | 0.25 | 0.18 | 0.26 | 0.11 | 0.08 |
|  | Correlation ↑ | 0.68 | 0.84 | 0.88 | 0.92 | 0.91 |
| Bei Lake | MAE ↓ | 0.18 | 0.19 | 0.19 | 0.21 | 0.21 |
|  | RMSE ↓ | 0.18 | 0.12 | 0.21 | 0.19 | 0.27 |
|  | Correlation ↑ | 0.60 | 0.77 | 0.88 | 0.93 | 0.90 |
| Sanbao Lake | MAE ↓ | 0.18 | 0.19 | 0.21 | 0.20 | 0.21 |
|  | RMSE ↓ | 0.12 | 0.22 | 0.19 | 0.21 | 0.21 |
|  | Correlation ↑ | 0.76 | 0.79 | 0.89 | 0.91 | 0.92 |
| Chedun Lake | MAE ↓ | 0.19 | 0.19 | 0.21 | 0.20 | 0.20 |
|  | RMSE ↓ | 0.22 | 0.21 | 0.23 | 0.19 | 0.19 |
|  | Correlation ↑ | 0.55 | 0.83 | 0.88 | 0.95 | 0.88 |
| Ducang Lake | MAE ↓ | 0.18 | 0.21 | 0.19 | 0.20 | 0.20 |
|  | RMSE ↓ | 0.18 | 0.22 | 0.19 | 0.19 | 0.26 |
|  | Correlation ↑ | 0.68 | 0.82 | 0.87 | 0.93 | 0.91 |
| ChuanJ Lake | MAE ↓ | 0.22 | 0.19 | 0.21 | 0.20 | 0.20 |
|  | RMSE ↓ | 0.19 | 0.18 | 0.19 | 0.14 | 0.15 |
|  | Correlation ↑ | 0.78 | 0.80 | 0.87 | 0.88 | 0.93 |
| JiuGC Lake | MAE ↓ | 0.22 | 0.29 | 0.21 | 0.21 | 0.11 |
|  | RMSE ↓ | 0.24 | 0.18 | 0.21 | 0.21 | 0.11 |
|  | Correlation ↑ | 0.53 | 0.82 | 0.88 | 0.92 | 0.88 |
| Jinji Lake | MAE ↓ | 0.21 | 0.21 | 0.11 | 0.10 | 0.10 |
|  | RMSE ↓ | 0.22 | 0.19 | 0.11 | 0.09 | 0.11 |
|  | Correlation ↑ | 0.52 | 0.80 | 0.89 | 0.85 | 0.95 |
| Shenshan Lake | MAE ↓ | 0.21 | 0.19 | 0.19 | 0.20 | 0.20 |
|  | RMSE ↓ | 0.18 | 0.21 | 0.11 | 0.21 | 0.20 |
|  | Correlation ↑ | 0.59 | 0.80 | 0.90 | 0.90 | 0.92 |

Note: For each lake and metric (MAE, RMSE, and Pearson correlation), the best-performing method is highlighted in blue. Arrows indicate the desired optimization direction: ↓ denotes lower is better, ↑ denotes higher is better.
